# Validation of an electronic algorithm to identify appropriate antibiotic use for community-acquired pneumonia in children

**DOI:** 10.1017/ash.2023.381

**Published:** 2023-09-29

**Authors:** Kathleen Chiotos, Robert Grundmeier, Didien Meyahnwi, Lauren Dutcher, Ebbing Lautenbach, Melinda Neuhauser, Keith Hamilton, Anne Jaskowiak, Leigh Cressman, Julia Szymczak, Brandi Muller, Jeffrey Gerbe

## Abstract

**Background:** Community-acquired pneumonia (CAP) is a common indication for antibiotic use in hospitalized children and is a key target for pediatric antimicrobial stewardship programs (ASPs). Building upon prior work, we developed and refined an electronic algorithm to identify children hospitalized with CAP and to evaluate the appropriateness of initial antibiotic choice and duration. **Methods:** We performed a cross-sectional study including children 6 months to 17 years hospitalized for CAP between January 1, 2019, and October 31, 2022, at a tertiary-care children’s hospital. CAP was defined electronically as an *International Classification of Disease, Tenth Revision* (ICD-10) code for pneumonia, a chest radiograph or chest computed tomography scan (CT) performed within 48 hours of admission, and systemic antibiotics administered within the first 48 hours of hospitalization and continued for at least 2 days. We applied the following exclusion criteria: patients transferred from another healthcare setting, those who died within 48 hours of hospitalization, children with complex chronic conditions, and those with intensive care unit stays >48 hours. Criteria for appropriate antibiotic choice and duration were defined based on established guidelines. Two physicians performed independent medical record reviews of 80 randomly selected patients (10% sample) to evaluate the performance of the electronic algorithm in (1) identifying patients treated for clinician-diagnosed CAP and (2) classifying antibiotic choice and duration as appropriate. A third physician resolved discrepancies. The electronic algorithm was compared to this medical record review, which served as the reference standard. **Results:** Of 80 children identified by the electronic algorithm, 79 (99%) were diagnosed with CAP based on medical record review. Antibiotic use was classified as the appropriate choice in 75 (94%) of 80 cases, and appropriate duration in 16 (20%) of 80 cases. The sensitivity of the electronic algorithm for identifying appropriate initial antibiotic choice was 94%; specificity could not be calculated because no events of inappropriate antibiotic choice were identified based on chart review. The sensitivity and specificity for determining appropriate duration were 88% and 97%, respectively (Table 1).

**Conclusions:** The electronic algorithm accurately identified children hospitalized with CAP and demonstrated acceptable performance for identifying appropriate antibiotic choice and duration. Use of this electronic algorithm may improve the efficiency of stewardship activities and could facilitate alignment with updated accreditation standards. Future studies validating this algorithm at other centers are needed.

**Figure:**
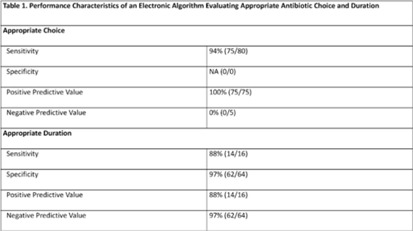

**Disclosures:** None

